# Groin Hernias in Women—A Review of the Literature

**DOI:** 10.3389/fsurg.2019.00004

**Published:** 2019-02-11

**Authors:** Ferdinand Köckerling, Andreas Koch, Ralph Lorenz

**Affiliations:** ^1^Department of Surgery and Center for Minimally Invasive Surgery, Academic Teaching Hospital of Charité Medical School, Vivantes Hospital, Berlin, Germany; ^2^Hernia Center Cottbus, Cottbus, Germany; ^3^Hernia Center 3+CHIRURGEN, Berlin, Germany

**Keywords:** groin hernia, women, femoral hernia, emergency, inguinal hernia

## Abstract

**Background:** To date, there are few studies and no systematic reviews focusing specifically on groin hernia in women. Most of the existing knowledge comes from registry data.

**Objective:** This present review now reports on such findings as are available on groin hernia in women.

**Materials and Methods:** A systematic search of the available literature was performed in September 2018 using Medline, PubMed, Google Scholar, and the Cochrane Library. For the present analysis 80 publications were identified.

**Results:** The lifetime risk of developing a groin hernia in women is 3–5.8%. The proportion of women in the overall collective of operated groin hernias is 8.0–11.5%. In women, the proportion of femoral hernias is 16.7–37%. Risk factors for development of a groin hernia in women of high age and with a positive family history. A groin hernia during pregnancy should not be operated on. The rate of emergency procedures in women, at 14.5–17.0%, is 3 to 4-fold higher than in men and at 40.6% is even higher for femoral hernia. Therefore, watchful waiting is not indicated in women. During surgical repair of groin hernia in females the presence of a femoral hernia should always be excluded and if detected should be repaired using a laparo-endoscopic or open preperitoneal mesh technique. A higher rate of chronic postoperative inguinal pain must be expected in females.

**Conclusion:** Special characteristics must be taken into account for repair of groin hernia in women.

## Introduction

To date, there are few studies and systematic reviews focusing specifically on groin hernia in women (1). Most of the existing knowledge comes from registry and national database analyses (1). All guidelines for the repair of groin hernia point to the special characteristics in women and these are taken into account in special treatment recommendations (1–6). But even when the guidelines for repair of groin hernia in females are observed, the outcome appears to be less favorable than in men (7). Therefore, further studies are urgently needed to better evaluate the factors impacting the outcome of groin hernia repair in women. To that effect, this review now aims to collate all existing findings on groin hernia in women.

## Materials and Methods

A systematic search of the available literature was performed in October 2018 using Medline, PubMed, Google Scholar, and the Cochrane Library. The following search terms were used: “Inguinal hernia and women,” “Groin hernia and women,” “Femoral hernia and women,” “Inguinal hernia and female,” “Groin hernia and female,” “Female hernia,” “inguinal hernia and gender,” “groin hernia and gender.”

**Figure 1:**
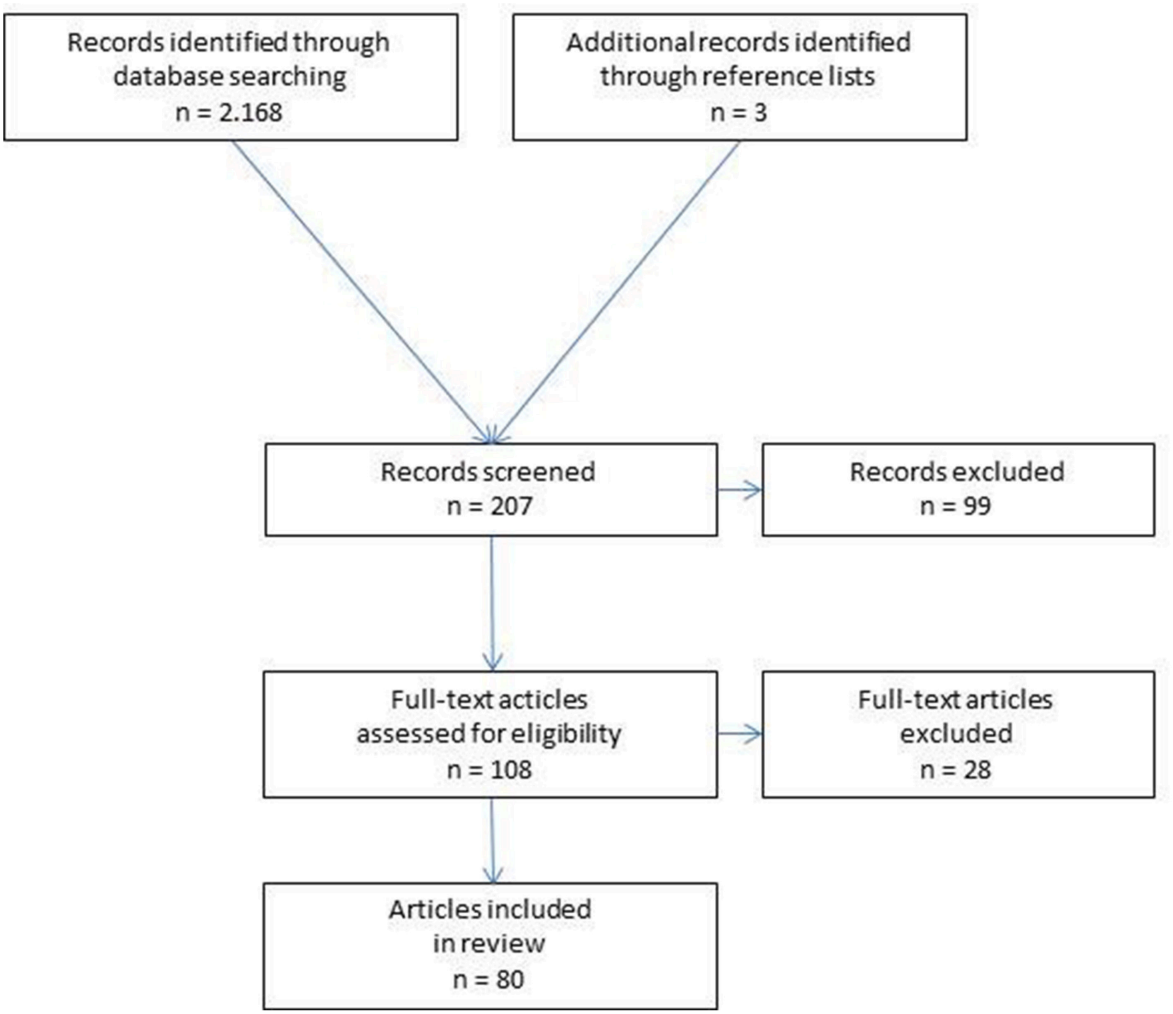
Flowchart of study inclusion.

The abstracts of 207 publications were checked. Ninety nine abstracts demonstrated no relevance for this review and were excluded. Assessment of eligibility of the 108 full-text articles lead to exclusion of 28. For the present analysis 80 publications were identified as relevant (Figure 1). According to the Prisma guidelines the characteristics and findings of the included studies are presented (8).

## Results

### Lifetime Risk

Groin hernias are rare in women compared to men. The lifetime risk of developing a groin hernia is given in the literature as 27–42% for men and 3–5.8 % for women (9–12).

### Proportion of Women With Operated Groin Hernias

The proportion of women in the overall patient collective of operated groin hernias in registries and administrative data is 8.0–11.5% (12–16). Hence, groin hernia repairs are conducted 8 to 10-fold more often in men than in women (1).

### Proportion of Femoral Hernias in Women

The rate of femoral hernias in women is higher than in men (1). In the Swedish Hernia Registry out of 79,534 elective groin hernia repairs in men, the proportion of femoral hernias was 0.7% and in 5,733 women it was 16.7%, thus amounting to an overall rate of 1.8% (17). It must, however, be pointed out here that in women in *n* = 1,339/6,895 (19.4%) and in men in *n* = 35,468/83,753 (42.3%), groin hernia repairs were performed with the Lichtenstein technique. Since in the Lichtenstein technique the transversalis fascia is not routinely opened, the rate of femoral hernias in men and women may by all means be higher. In the Danish Hernia Database out of the recorded 148,277 groin hernia repairs, 3,970 were primary femoral hernias, constituting a proportion of 2.7% (18). In the American College of Surgeons—National Surgical Quality Improvement Program database, femoral hernias accounted for 2.8% of initial groin hernias and 18.9% of all groin hernias in females (19). Elective and emergent femoral hernia repairs constitute roughly 2–4% of all groin hernia repairs (1). As in the Swedish Hernia Registry, a study at the Shouldice Hospital likewise identified a proportion of femoral hernias of 17% (20).

Thanks to the diagnostic superiority of laparo-endoscopic techniques, the incidence of femoral hernias has been as high as 23.54–37% for women and 3% for men (21, 22).

### Lipomas of the Round Ligament

Lipomas of the round ligament occur with a significant incidence (23). They can cause hernia-type symptoms in the absence of a true hernia (23). They should be considered in female patients with groin pain and normal examination results (23). They can be easily overlooked and lead to an unsatisfactory result (23).

### Risk Factors

In an anatomical study of the inguinal region concerning anatomic differences in men and women with reference to hernia formation, there was a significant difference between the diameters of the internal rings, which were almost twice as large in men, while the width of the rectus abdominis muscle was significantly greater in women (24). These anatomic differences may possibly explain why in women the lifetime risk for the development of a groin hernia may be significantly lower than in men.

Another risk factor is high age (25, 26). In women the incidence rate per 100,000 person years increases from 15.1 in 18–29 year olds to 148.1 in 80–89 year olds (12).

Another independent risk factor is a positive family history (25, 27). Inguinal hernias are hereditary with a complex multifactorial inheritance pattern (25).

Surprisingly, a population-based study from the USA identified a lower incidence of groin hernias in patients with a body mass index (BMI) of 30–34.9 and ≥35 compared with persons of normal weight (BMI <25) and overweight patients (BMI 25–29.9) (28). That relationship was confirmed once again by data from the Swedish Hernia Registry and by administrative data from the USA (29, 30). The relationship probably has a risk of bias, since it is easier to detect an inguinal hernia at lower BMI (25). Inguinal hernias are common in patients with connective tissue disorders (25).

Multiple deliveries were not associated with inguinal hernia in females (27).

Nor did smoking have any impact (25).

Physical activity appears to have a negative effect by the increase of the intra-abdominal pressure (25).

### Preoperative Diagnostics

In a systematic review and meta-analysis of diagnosis of clinically occult groin hernias, 16 of the included studies demonstrated that ultrasound was able to reliably distinguish between inguinal and femoral hernia (31).

Accuracy may strongly depend on the examiner's skills (31). Based on current evidence, the sensitivity, specificity, and negative predictive value of ultrasound in detecting clinically occult groin hernia cannot reliably be determined (31), as its diagnostic accuracy is reduced in the absence of any clinically palpable hernia (32). When there still is diagnostic uncertainty, further investigation with magnetic resonance imaging should be considered to exclude alternative pathology (33).

### Groin Hernia and Pregnancy

From an overall collective of 20,714 pregnant women, 25 (0.12%) had a primary groin hernia (34). None of the pregnant women had to undergo elective or emergency groin hernia surgery and all women gave uncomplicated birth (34). During a 4.4 years follow-up, four patients (0.02%) underwent elective groin hernia operation (34). The authors recommend a watchful waiting strategy during pregnancy in women with suspected groin hernia (34). In a prospective clinical study, 18 pregnant women with clinically suspected groin hernias showed in a gray-scale and color Doppler sonography large varicose veins along the round ligament (35). All women gave uncomplicated birth to single children (35). The authors concluded that surgical exploration of the groin during pregnancy must be avoided (35). According to a recommendation in the new international Guidelines of the HerniaSurge Group watchful waiting is suggested in pregnant females with groin swelling (1).

### Risk of Emergency Surgery

In the population-based study from the USA the proportion of groin hernia emergency procedures was 3.8%, with 3.0% in men and 14.5% in women (36). In the Swedish Hernia Registry, the emergency procedure rate was 5.1% in men and 17.0% in women (17, 37). As such, the emergency procedure rate was 3 to 4-fold higher in women than in men. Compared with elective groin hernia operations, patients undergoing emergency procedures are older, obese, have a higher ASA score and more femoral hernias or a recurrence (36). In the presence of a femoral hernia, the risk of an emergency procedure in men increases from 3.0–5.1% to 28.1% and in women from 14.5–17.0 to 40.6% (38). Most femoral hernias present incarcerated in older, female patients (19).

Furthermore, it was demonstrated that patients with femoral hernias were often completely asymptomatic up to the time of emergency surgery (39). Likewise, a British study revealed that 81.5% of patients who had undergone an emergency procedure for incarcerated femoral hernia first presented with symptoms to the general practitioner within the week prior to hospital admission (40).

Since the risk of emergency procedure in women with a groin hernia is 3 to 4-fold higher than in men, rising to 40% in those with a femoral hernia, a “watchful waiting” concept cannot be justified in women even in the absence of symptoms. Therefore, the international guidelines of the HerniaSurge Group feature a strong upgraded recommendation for timely elective repair of groin hernias in women (1).

### Outcome of Emergency Surgery

Compared with 90,777 elective groin hernias operations in men and 6,656 in women with a mortality of 0.1%, the mortality rate following emergency procedures was 3.7% in women and 2.7% in men (37). After femoral hernia operation, the mortality risk was increased 7 to 10-fold for men and women (37, 38). Since the incidence of groin and femoral hernia was highest in the age group ≥65 years (41) and patients undergoing emergency procedure belong predominantly to that age group and generally have serious comorbidities, the mortality risk is no doubt multifactorial (41). Femoral hernias present more commonly incarcerated in patients with significant comorbid diseases and are associated with significantly increased rates of return to the operation theater, and mortality (19).

### Techniques of Groin Hernia Repair in Women

The laparo-endoscopic techniques (total extraperitoneal patch plasty = TEP and transabdominal preperitoneal patch plasty = TAPP) have diagnostic advantages in identifying femoral hernias (1). Single center studies have continually reported positive outcomes for the laparo-endoscopic technique in repair of groin hernia in women (21, 22, 42–45). Accordingly, all guidelines recommend the TEP and TAPP laparo-endoscopic techniques for repair of groin hernia in women (1–6). The preperitoneal mesh placement in TEP and TAPP also provides for coverage of the femoral hernias (1–6). However, the treating surgeon must have appropriate experience with TEP or TAPP (1).

Thairu et al. reported a recurrence rate of 2.8% for 37 women operated on with a non-mesh open repair technique (nylon darn repair) (46). The transversalis fascia is not split open in the darn repair (47). In the concept of the nylon darn repair the posterior wall of the inguinal canal is reinforced tension-free by synthetic suture material woven between the myoaponeurotic arch and the inguinal ligament (47).

Alimoglu et al. (48) treated 79 patients with femoral hernia, including emergency cases, in McVay technique, as Cooper ligament hernioplasty without mesh. Recurrences occurred in 2.4% of the patients (48).

The Shouldice Hospital reported on 256 patients with a femoral hernia with 225 completing 5 years of follow-up after repair (49). Concurrent inguinal hernias were found in 115 patients (51%), and 41 (18.2%) had a previous inguinal hernia repair. A complete groin tissue repair was performed in 120 patients and a preperitoneal mesh repair in 78, with the remaining having infra-inguinal mesh repair (49). Fifty six percentage of the patients were female. The overall recurrence rate was 3.1% (49). The authors concluded that femoral hernias can be repaired electively with a tissue-based or a preperitoneal mesh technique (49).

Babar et al. (50) found no recurrence in women after modified Nyhus-Condon femoral hernia repair with mesh. The mesh is placed in the preperitoneal space (50).

Kark et al. (51) found in a consecutive series of 255 women with primary groin hernias and 20% femoral hernias that Lichtenstein repair was easier than in men, and as effective (51). Female hernias were approached through an incision made over the swelling (51). A cone of polypropylene mesh was inserted into the femoral canal and anchored by three non-absorbable sutures (51). They found no recurrence in a median follow-up of 44.5 months (51).

Three studies (52–54) present positive results in the treatment of femoral hernias with the use of mesh plug technique. In a prospective randomized trial comparing preperitoneal with plug mesh repair in femoral hernias the recurrence rate, the rate of foreign body sensation and the seroma rate was lower for preperitoneal herniorrhaphy (53). In the new international guidelines of the HerniaSurge Group plug and patch techniques are no longer recommended (1).

### Distribution of Groin Hernia Repair Techniques for Women in Registries

In the Swedish Hernia Registry of the 9,756 elective groin hernia repairs in women, 60.6% were conducted with an open anterior mesh, 23.0% with suture, 11.2% with laparo-endoscopic and 5.2% with an open posterior mesh technique (16). In the Danish Hernia Database of the 13,945 primary groin hernia repairs in women a distinction was made between inguinal and femoral hernias (55). Groin hernia repair was carried out in 44% of cases with the Lichtenstein, 40% with laparo-endoscopic, 10% with open non-mesh and 6% with another open mesh technique (55). Femoral hernia repair was performed in 40% of cases with the laparo-endoscopic, in 34% with plug and in 26% with the McVay technique, with and without mesh.

### Outcome of Elective Groin Hernia Repair in Women

#### Recurrence

Of the 13,945 primary groin hernia operations in women with a median follow-up time of 8.8 years, 649 (4.7%) patients had a recurrence requiring reoperation (55). The cumulative reoperation rates were lower after laparoscopic repair compared with the open techniques, for both inguinal hernias (1.8% vs. 6.3%; *p* < 0.001) and femoral hernias (2.2% vs. 5.5%; *p* = 0.005) (55). After laparoscopic repair, 25% of inguinal hernias recurred as femoral hernias, compared with 47% after Lichtenstein (*p* < 0.001) (55). Direct inguinal hernias and femoral hernias had higher risk of reoperation for recurrence after open repair compared with indirect inguinal hernias (55). The reoperation rates were similar for laparo-endoscopic repair of hernia subtypes during primary groin hernia repair (55).

In an analysis of data in the Swedish Hernia Registry relating to 6,895 groin hernia repairs in women and 83,753 in men, femoral hernias were found during the recurrence operations in 41.6% of women and only in 4.6% of men (17). In the primary operation hernias had been classified as either direct or indirect groin hernias. Multivariable analysis revealed that the use of laparo-endoscopic techniques for the primary operation reduced the recurrence risk compared with the Lichtenstein and Shouldice operations (17). Likewise, in the Danish Hernia Database with 3,696 female inguinal hernia repairs, a 41.5% femoral hernia detection rate was identified during reoperations for hernia recurrence. In the male comparative collective the rate was only 5.4% (56, 57). The reoperation rate was independent of the type of surgical repair (57). These femoral recurrences occurred earlier than inguinal recurrences suggesting that they were possibly femoral hernias that had been overlooked during the primary operation (56). In a systematic review and meta-analysis of observational studies concerning patient-related risk factors for recurrence after inguinal hernia repair, five studies with 284,898 procedures in 284,898 persons were included (17, 57–60). The meta-analysis found female sex to be a risk factor for recurrence (61). The authors believe that higher recurrence rates in females could be attributable to femoral hernias being overlooked during the primary operation (61). In a multivariate adjusted analysis of 5,893 female groin hernias, it was found that medial inguinal hernia at the primary operation was a substantial risk factor for recurrence with a hazard ratio of 3.1 (CI 95%; 2.4–3.9) compared with lateral inguinal hernia of primary operation (*p* < 0.001), and that laparoscopic operation delivers a lower risk of recurrence with a hazard ratio of 0.57 (CI 95% 0.43–0.75) compared with the Lichtenstein technique (*p* < 0.001) (62).

Based on these registry data, in one review of the causes of recurrent groin hernia the increased rate of femoral recurrence in female patients following open primary operations was interpreted as overlooked femoral hernias (63). The surgeon had neglected to look for a femoral hernia during the open-technique primary groin hernia operation (63). The overlooked femoral hernias later become symptomatic (63). That is particularly true for the open techniques such as the Lichtenstein operation when the transversalis fascia is not routinely opened (62).

In a further study of 3,970 primary femoral hernias from the Danish Hernia Database (39.2% emergency and 60.8% elective procedures), multivariable analysis revealed that the laparoscopic technique had a reduced risk of reoperation due to recurrence (hazard ratio: 0.33; 95% CI, 0.09–0.95) compared with the open operation (18).

The National Surgical Quality Improvement Program (NSQIP) database of the American College of Surgeons has revealed that for 6,649 femoral hernia repairs in women the proportion of femoral hernia recurrence declined from of 14.0% in 2005 to 6.6% in 2014 (64). That shows that awareness of the possible existence of a femoral hernia has grown and that surgeons have learned how to control the problem. That is done either by opting for the TEP and TAPP laparo-endoscopic techniques or an open technique while opening the transversalis fascia and looking specifically for femoral hernias (65). Appropriate repair can then be performed using a TEP or TAPP laparo-endoscopic or open technique with preperitoneal mesh implant to cover the femoral hernia (48, 50, 53, 66). A Cochrane review has once again identified a reduction in the recurrence rate through the use of a mesh method for repair of inguinal and femoral hernias (67). In view of the superior diagnostic capability of laparo-endoscopic techniques, the international guidelines of the HerniaSurge Group therefore recommend the totally extraperitoneal patch plasty (TEP) or the transabdominal preperitoneal patch plasty (TAPP) for groin and femoral hernia repair in women (1). But data are also available in the meantime showing that if a femoral hernia is reliably ruled out, the Shouldice technique, too, can be used for repair of groin hernia in selected female patients (68, 69). However, if a femoral hernia is detected after opening the transversalis fascia in the Shouldice operation, then using a tailored approach changeover to preperitoneal mesh placement would have to be done (48, 50, 53, 66).

#### Postoperative Pain

Women have higher postoperative pain than men (70). Female gender is a strong risk factor for chronic postoperative inguinal pain (CPIP) (71–73). In a comparative study in elective inguinal hernia repair in TAPP technique, women experienced significantly more pain, discomfort, and fatigue (7).

The risk of complicated presentation and unfavorable outcome in patients with groin hernia is significant in female sex (74).

In a median follow-up time of 4.7 years after femoral hernia repair with a proportion of 72% women, some degree of pain during the previous week was reported by 24.2% of patients (75). Pain that interfered with daily activities was found in 5.5% of patients (75).

#### Quality of Life

In a comparative study of laparoscopic vs. open repair of femoral hernias with a proportion of 60.3% women, no difference was found in the operative times, long-term outcomes, or quality of life (76).

#### Intestinal Obstruction

Following inguinal and femoral hernia repair documented in the Swedish Hernia Registry, univariate Cox analyses revealed that female sex and femoral hernia were significant risk factors for postoperative intestinal obstruction (77).

### Special Aspects

#### Transection of the Round Ligament in Laparo-Endoscopic Repair

In an electronic questionnaire sent to all surgeons in Denmark the aim was to investigate how often a national cohort of experienced groin hernia surgeons transected the round ligament in laparo-endoscopic (TAPP, TEP) groin hernia repair (78). The response rate was 86%. The round ligament was transected in 49% of all laparo-endoscopic procedures during the past 12 months (78). The consequences of transecting the round ligament are not well-understood (78).

In a single center study the clinical data of 316 female patients with 341 hernias were retrospectively analyzed. 274 TAP and 67 TEP procedures have been performed (79). Round ligament of uterus were preserved in 152 patients and transected in 162. The preservation group requires longer operation time and trickier surgical technique (79).

#### Inguinal Endometriosis

Inguinal endometriosis is a rare disease and often misdiagnosed (80). Typically, they presented with a right-sided swelling in the groin (80). Surgeons should be aware of this disease in fertile women with a lump in the groin (80).

## Discussion

The aim of this review is to collate all existing findings on groin hernia in women. Most of the existing knowledge comes from analyses of registries and national data bases. There are very few randomized controlled trials or systematic reviews. All guidelines for the repair of groin hernia point to be special characteristics in women taken into account for special treatment recommendations (1–6).

The lifetime risk for development of a groin hernia in women is 3–5.8% compared to 27–42% in men. Groin hernia repairs are therefore conducted 8 to 10-fold more often in men than in women. In the literature the proportion of women in the overall collective of operated groin hernias is 8.0–11.5% (12–16). The proportion of femoral hernias in all groin hernias in women is 16.7–37% (17–22). Due to the diagnostic superiority of the laparo-endoscopic techniques TEP and TAPP, the incidence of femoral hernias has been as high as 23.5–37% for women and 3% for men (21, 22). Lipomas of the round ligament occur in women with a significant incidence and should be treated when symptomatic (23).

Risk factors for development of groin hernia in women are high age, positive family history, connective tissue disorders and physical activities with increase of intraabdominal pressure (25). The low incidence of groin hernias in patients with a higher Body Mass Index has probably a risk of bias, since it is easier to detect an inguinal hernia at lower Body Mass Index (25).

The use of ultrasound and magnetic resonance imaging is able to distinguish between inguinal and femoral hernias (31–33).

Groin hernias should as a rule not be operated on during pregnancy (34, 35).

The rate of emergency procedures in women at 14.5–17.0% is 3 to 4-fold higher than in men (17, 36, 37). In the presence of a femoral hernia, the risk of emergency procedure in women rises to 40.6% (38). Patients with femoral hernias are often asymptomatic up to the time of the emergency procedure (39, 40). Most femoral hernias present incarcerated in older female patients (19). Therefore, watchful waiting is not indicated in women (1).

The mortality risk following a femoral hernia emergency procedure is 7 to 10-fold higher (37, 38). As the patients undergoing emergency procedure belong predominantly to the higher age group and generally have serious comorbidities, the mortality risk is multifactorial (41). Femoral hernias present more commonly incarcerated in patients with significant comorbid diseases and are associated with significantly increased rates of reoperations due to surgical complications, and mortality (19).

During surgical treatment of a groin hernia in women, a femoral hernia should always be reliably ruled out (1). A femoral hernia in women can be optimally repaired with a laparo-endoscopic TEP and TAPP (1) or open preperitoneal mesh placement technique (49, 53).

If a femoral hernia is not reliably ruled out or not appropriately treated, a high early recurrence rate must be expected in women (17, 55–61). The authors of a systematic review and meta-analysis believe that higher recurrence rates in females could be attributable to femoral hernias being overlooked during the primary operation (61). This is particularly true for the open techniques such as a Lichtenstein operation, when the transversalis fascia is not routinely opened (62).

Even with appropriate treatment of groin hernia in women, a higher rate of chronic postoperative inguinal pain must be expected (70–75). In a comparative study in elective inguinal hernia repair in TAPP technique, women experienced significantly more pain, discomfort, and fatigue (7).

In conclusion many special characteristics for groin hernia repair in women must be taken into account. All guidelines include specific recommendations for the diagnosis and treatment of female groin hernias. Despite consideration of the guidelines, the outcome appears less favorable in women than in men. Therefore, groin hernia repair in women should be performed by an experienced surgeon being aware of all specific aspects and consideration of the guidelines.

## Author Contributions

FK: literature search, literature analyses, publication concept, and publication draft; RL: literature search, literature analyses, publication concept, and critical review of the publication draft; AK: literature search, literature analyses, publication concept, critical review of the publication draft.

### Conflict of Interest Statement

The authors declare that the research was conducted in the absence of any commercial or financial relationships that could be construed as a potential conflict of interest.
